# Risk of exposure to potential vector mosquitoes for rural workers in Northern Lao PDR

**DOI:** 10.1371/journal.pntd.0005802

**Published:** 2017-07-25

**Authors:** Julie-Anne A. Tangena, Phoutmany Thammavong, Steve W. Lindsay, Paul T. Brey

**Affiliations:** 1 Department of Medical Entomology & Biology of Disease Vectors, Institut Pasteur du Laos, Vientiane, Lao PDR; 2 School of Biological and Biomedical Sciences, Durham University, Durham, United Kingdom; National Center for Atmospheric Research, UNITED STATES

## Abstract

**Background:**

One major consequence of economic development in South-East Asia has been a rapid expansion of rubber plantations, in which outbreaks of dengue and malaria have occurred. Here we explored the difference in risk of exposure to potential dengue, Japanese encephalitis (JE), and malaria vectors between rubber workers and those engaged in traditional forest activities in northern Laos PDR.

**Methodology/Principal findings:**

Adult mosquitoes were collected for nine months in secondary forests, mature and immature rubber plantations, and villages. Human behavior data were collected using rapid participatory rural appraisals and surveys. Exposure risk was assessed by combining vector and human behavior and calculating the basic reproduction number (R_0_) in different typologies. Compared to those that stayed in the village, the risk of dengue vector exposure was higher for those that visited the secondary forests during the day (odds ratio (OR) 36.0), for those living and working in rubber plantations (OR 16.2) and for those that tapped rubber (OR 3.2). Exposure to JE vectors was also higher in the forest (OR 1.4) and, similar when working (OR 1.0) and living in the plantations (OR 0.8). Exposure to malaria vectors was greater in the forest (OR 1.3), similar when working in the plantations (OR 0.9) and lower when living in the plantations (OR 0.6). R_0_ for dengue was >2.8 for all habitats surveyed, except villages where R_0_≤0.06. The main malaria vector in all habitats was *Anopheles maculatus* s.l. in the rainy season and *An*. *minimus* s.l. in the dry season.

**Conclusions/Significance:**

The highest risk of exposure to vector mosquitoes occurred when people visit natural forests. However, since rubber workers spend long periods in the rubber plantations, their risk of exposure is increased greatly compared to those who temporarily enter natural forests or remain in the village. This study highlights the necessity of broadening mosquito control to include rubber plantations.

## Author summary

Rapid economic development in South-East Asia has resulted in a high demand for rubber, leading to the felling of natural forest and the expansion of rubber plantations. Hundred-thousands of people work in these man-made forests throughout the region, with some studies showing a higher risk of vector-borne diseases for rubber workers compared to typical village populations. In this study we assessed the risk of exposure to vector mosquitoes in relation to different typologies of human behavior. Whilst the highest risk of vector-borne diseases is in natural forest, those living and working in the rubber plantations are at higher risk of dengue and lower risk of malaria vector exposure than villagers that stay in the village. As dengue is endemic in our study area and malaria is not, rubber workers should be encouraged to live in the villages instead of plantations. Furthermore, vector-borne disease control in Lao PDR should broaden from its current focus on villages to include outdoor protection in both rubber plantations and forests, using larval control and personal protection methods.

## Introduction

Today we have entered the Anthropocene epoch, in recognition of the major impact human beings have on the environment [1]. Many of the changes in land use and climate are also likely to increase the risk of vector-borne diseases [2–6]. One of the largest environmental changes in South-East Asia (SEA) has been the rapid expansion of rubber plantations. Natural rubber, obtained as latex from the rubber tree *Hevea brasiliensis* (Willd. ex A Juss.), provides 42% of the global rubber [7, 8]. In 2010 rubber plantations covered 9.2 million ha in SEA [9], supplying more than 90% of the global demand for natural rubber [10]. Stimulated by the high profitability of this crop, the area cultivated for mature rubber in Lao PDR increased rapidly from 900 ha in 2010 to 147,500 ha in 2015 [11]. This is likely to increase to 342,400 ha of mature rubber plantations in the next decade, employing over 100,000 people [11]. Although rubber cultivation is decreasing with the slowdown in the Chinese economy, an estimated four and a half to six million workers will be needed to tap the mature rubber trees in the region in the next decade [12]. Outbreaks of mosquito-borne diseases such as malaria, dengue, and chikungunya have been reported in rubber plantations [13–16]. It has been suggested that rubber workers in SEA are at increased risk of malaria, as plantation workers tap latex at night when malaria vectors are active [13]. With the high number of migrant workers in the rubber plantations, there is fear that these plantations may aid the spread and increase the incidence of mosquito-borne diseases in the region.

Surprisingly little work has been done to assess the importance of rubber plantations as a nidus for the transmission of mosquito-borne diseases in SEA. In this study we investigated the risk of exposure to dengue, Japanese encephalitis (JE), and malaria vectors in relation to different patterns of behavior or typologies commonly represented in this part of northern Lao PDR, in order to understand which behaviors put people most at risk from mosquito-borne diseases.

## Methods

### Study sites

The adult mosquito sampling and behavioral studies were conducted in Thinkeo (19°41’02.13”N 102°07’05.49”E), Silalek (19°37’02.80”N 102°03’05.70”E), and Houayhoy (19°33’03.22”N 101°59’42.42”E) in Xieng-Ngeun and Nane district, Luang Prabang province. In each study site four common habitats were selected: secondary forests, mature rubber plantations, immature rubber plantations, and villages. The secondary forests were young forests consisting of young small trees with a high density of undergrowth. The mature rubber plantations were those where >70% of the trees were tapped for latex and the immature rubber plantations consisted of rubber trees less than five years old which have not been tapped for latex. The rural villages were small linear settlements of about 150 to 200 bamboo and cement houses. The risk of mosquito-borne disease is highest during the rainy season from May to October. Dengue and JE cases are relatively common, but according to data from Xieng Ngeun and Nane district health centers, malaria has not been locally-transmitted in our study area, with one to five malaria cases imported into the districts every year.

### Adult mosquito sampling

Routine entomological measurements were made monthly for nine months from July to November 2013 and in February, March, May and July 2014. A detailed description of the mosquito species collected in the different habitats during the adult mosquito sampling is described in [17]. A total of 78 human subjects gave written informed consent to participate and collect mosquitoes using the human-baited double bed net (HDN) trap [18]. This trap consists of a person on a bamboo bed (30 cm high x 230 cm long x 100 cm wide) covered by two untreated bed nets (small: 97 cm high x 200 cm long x 100 cm wide, mesh size 1.5 mm; large 100 cm high x 250 cm long x 150 cm wide, mesh size 1.5 mm). The internal net protects the occupant from mosquito bites, whilst the outer large net is raised off the ground and traps mosquitoes coming to feed. Mosquitoes were collected outdoors from between the nets at hourly intervals during the day and night. A total of 36 HDN traps were used i.e. three HDN traps in each of the four different habitats. Mosquitoes were morphologically identified to species or species complex using stereo-microscopes and mosquito identification keys of Thailand [19].

### Human behavior studies

Daily and monthly activities of the rubber workers and villagers were described qualitatively in the three study sites in November 2013 using rapid participatory rural appraisals (PRA) [20]. All villagers and rubber workers from the study area were invited to participate in the discussions with a local translator present to facilitate the meeting. Participants were asked to complete timetables together, in which they recorded the intensity, from one to five, monthly and hourly according to their experience (one: very low, five: very high) for: rainfall, temperature, mosquito numbers, villagers feeling unwell and travel, including visits to secondary forest, latex tapping, collecting latex and rice production.

A further survey was carried out in June 2015, at the beginning of the rainy season, to collect information on the daily activities of the local population in the past 24 hours. The frequency of visits to the rubber plantations and the methods used to protect themselves from mosquito bites when outdoors was recorded. The study was conducted by a medical doctor fluent in the Lao language. For realistic representation of the different villages, 54 people per village were surveyed (power ω = 0.8, α = 0.05 and size effect of 0.5) [21]. Both studies were anonymous with no sensitive information collected.

### Basic reproduction number

Mosquito survival was assessed in all habitats in Thinkeo during the rainy season in July and August 2015. Two HDN traps were deployed in each habitat from 17.00–6.00 h. All *Anopheles* species previously identified as putative malaria vectors [22–29] and *Aedes albopictus* (Skuse), previously identified as a putative dengue vector in Lao PDR [30, 31], were dissected to determine parity [32]. The basic reproduction number (R_0_) for dengue and malaria was calculated in each habitat during both the rainy season (May-September) and dry season (October-April). R_0_ is calculated based on the Ross-Macdonald model and is an estimate of the number of new infections derived from one infective case in a habitat before the patient dies or is cured [33–35]. Values greater than one suggest that the pathogen would persist in an area if introduced, and values less than one indicate that the pathogen would become extinct.

R_0_ for dengue was calculated for *Ae*. *albopictus*, the only dengue vector in our study area, based on the following formulae (1) [36], using parameters in Table 1.

**Table 1:** Description of the parameters used for the dengue basic reproductive number model.

	Description	Formula/calculation
**a**	Frequency of the vector mosquito feeding on a person/day	a = C/x
**C**	Proportion of mosquitoes feeding on human blood instead of other animals	0.99 [37]
**x**	Gonotrophic cycle length, measured by the interval between blood meals taken	Conservative estimate of 4.5 days [38]
**r**	Rate of human recovery (1/number of days)	Four to five days [36, 39–41] So, 1/4.5
**ma**	Number of mosquito bites per person/day	Average number of mosquitoes collected per person/day during the adult mosquito sampling study
**μ**	Mortality rate of female mosquitoes	1- *p*
***p***	Daily survival probability of adult mosquitoes	A^1/X^
**A**	Average proportion of parous mosquitoes	Proportion parous from the mosquito survival data
**n**	Development days of virus in mosquito	Using graph [42] with Average T_dry_ in study area = 23.2 °C Average T_rain_ in study area = 23.3 °C
**b**	Proportion of female mosquitoes infective after taking infective blood meal	0.4 [36, 43]
**d**	Transmission from human to mosquito	0.4 [36, 43]

$$Dengue\;R_{0}=\frac{a}{r}ma^{2}e^{-\mu n}\frac{bd}{\mu }$$ (1)

The R_0_ for malaria was calculated for both *Plasmodium falciparum* and *Plasmodium vivax* malaria infections. We calculated the R_0_ for both parasites, since although 73% of all malaria infections in Lao PDR are due to *P*. *falciparum* [44], the last malaria outbreak recorded close to our study area was caused by *P*. *vivax*. The R_0_ was calculated for the primary malaria vectors *Anopheles maculatus* s.l., *An*. *minimus* s.l., and *An*. *dirus* s.l., using the following formula (2) [45, 46], with the description of parameters in Table 2.

**Table 2:** Description of the parameters used for the malaria basic reproductive number model.

	Description	Formula and calculation
**ma**	Number of mosquito bites per person/day	Average number of mosquitoes collected per person/day during the adult mosquito sampling study
**a**	Frequency of the vector mosquito feeding on a person/day	a = C/x
**C**	Proportion of mosquitoes feeding on human blood instead of other animals	1/3 proportion fed on human for *An*. *maculatus* s.l. *and An*. *minimus* s.l. [47] 2/3 proportion fed on human for *An*. *dirus* s.l.
**x**	Gonotrophic cycle length, measured by the interval between blood meals taken	2.35 days for *An*. *maculatus* s.l. [47, 48] Two days for rainy season and three days for dry season for *An*. *minimus* s.l.[49] Three days for *An*. *dirus* s.l. [23, 50]
**b**	Proportion of female mosquitoes developing parasites after taking an infective blood meal	Dependent on genetic and non-genetic determinants [51, 52], conservative estimate of 0.5 for all [53]
***p***	Daily survival probability of adult mosquitoes	A^1/X^
**A**	Average proportion of parous mosquitoes	Proportion parous from the mosquito survival data
**n**	Development days of parasite in mosquito (sporogonic cycle) using Moshkovsky's method	For *P*. *falciparum* the thermal sum required to complete parasite development is 111°C above 16°C. For *P*. *vivax* the thermal sum required to complete parasite development is 105°C above 14.5°C [54] Average T_dry_ in study area = 23.2 °C Average T_rain_ in study area = 23.3 °C
**r**	Rate of human recovery (1/number of days)	60 days, so 1/60 [55, 56]

$$Malaria\;R_{0}=\frac{ma^{2}bp^{n}}{-\ln(p)r}$$ (2)

### Analysis

The hourly mosquito sampling results were averaged for the nine months collection period to describe the daily activity of dengue, JE, and malaria vectors in the different habitats. The three PRA’s were summarized by taking the mean intensity of activities from the three appraisals. The study results were described as percentages. The exposure risk to the dengue vector *Ae*. *albopictus*, JE vector *Culex vishnui* s.l., and malaria vectors was assessed using several behavioral typologies. The daily activities of villagers and rubber workers were associated with vector mosquito exposure risk, using the entomological and human behavioral data. The basic reproductive numbers were calculated as described earlier and compared for the different habitats.

### Ethics

This study was approved by the Lao ethics committee (approval number 017/NECHR issued 21-04-2013) and the School of Biological and Biomedical Sciences Ethics Committee, Durham University (issued 25-07-2013).

## Results

### Mosquito sampling

**Fig 1:**
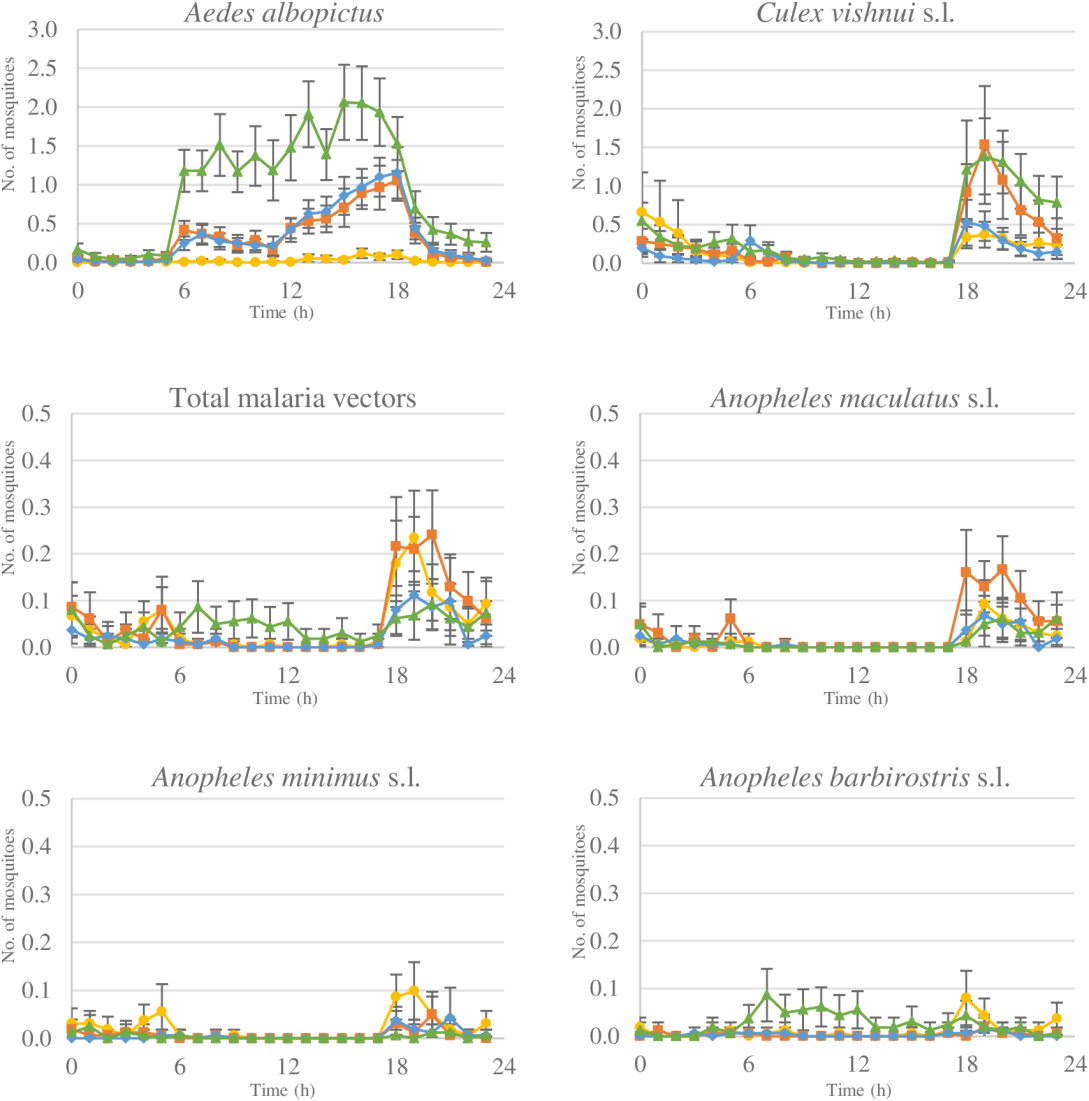
The average number of female mosquitoes collected per person/hour in the four different habitats (━▲━ secondary forests, ━♦━ mature plantations, ━■━ immature plantations, ━●━ villages) for *Aedes albopictus*, *Culex vishnui* s.l., total malaria vectors, *Anopheles maculatus* s.l., *Anopheles minimus* s.l., and *Anopheles barbirostris* s.l. during 24 hrs. All including 95% confidence interval.

During the adult mosquito sampling 24,927 females were collected. Of these 8,585 were *Aedes*, with 6,302 *Ae*. *albopictus*. The greatest numbers of *Ae*. *albopictus* were collected in the secondary forests with similar numbers in rubber plantation habitats (Fig 1). *Aedes albopictus* were active throughout the day, from 06.00 to 18.00 h. A total of 5,022 *Culex* were collected, of which 3,562 were *Cx*. *vishnui* s.l. *Culex vishnui* s.l. showed peak activity in the evening from 18.00 h to 20.00 h for all habitats (Fig 1). A total of 1,341 *Anopheles* mosquito species were collected, of which 661 were putative malaria vectors, including *An*. *maculatus* s.l. (n = 294), *An*. *barbirostris* s.l. (n = 170), *An*. *minimus* s.l. (n = 151 samples), and *An*. *dirus* s.l. (n = 46). Malaria vectors were collected in low numbers throughout the day and night. In the secondary forests *An*. *barbirostris* s.l. was mostly collected during the day and *An*. *maculatus* s.l. during the evening (Fig 1). In all the other habitats malaria vectors were generally collected between 18.00 to 21.00 h. The *An*. *dirus* s.l. mosquito samples collected in the different habitats showed similar behavior. About 67% of total *An*. *dirus* s.l. were collected between 18.00 and 22.00 h (30/46), with the remaining samples collected between 01.00 and 05.00 h. All data has been deposited in the Dryad repository http://dx.doi.org/10.5061/dryad.8nf05 [57].

### Human behavioral studies

Between 15 to 19 villagers, 16 to 60 years old, participated in a single two hour long PRA at each of the three study sites. During the rainy season (May to November) considerable time was spent cultivating rice, the staple food. Secondary forests were also visited during the rains, most frequently during daylight hours (05.00 h to 17.00 h; S1 Table), to collect food, wood, and other commodities. Occasionally the forests were visited at night to hunt small animals, like rodents and muntjacs. Rubber tapping also occurred in the rainy season with the trees tapped at night, between 02.00 h and 07.00 h, when latex flow is highest. Generally latex is collected from the latex collection cups in the morning from 07.00 h to 10.00 h. From 17.00 to 07.00 h usually most villagers were in the village to cook, clean, and sleep. Young children (< 14 years), villagers who did not have to work and elderly villagers (> 60 years) stayed in the village throughout the day. From December to February, when there was no farming, some villagers travelled to other parts of Lao PDR and abroad to find work (S2 Table).

A total of 162 participants were surveyed to identify their movement in the last 24 hrs, of which 8.6% (14/162) were rubber workers. Usually villagers 14 to 55 years old leave the village during the day from 07.00 h to 17.00 h; with 40% (65/162) working on the farm, 10% (17/162) going to high school, 5% (8/162) working in rubber plantations, 3% (5/162) going to the forest and 3% (4/162) visiting Luang Prabang, the provincial capital. The remaining 39% (63/162) stayed in the village. More than 91% (147/162) of villagers and rubber workers stayed in the village at night the day before the study was conducted. They generally slept from 20.00 h to 05.00 h. The remaining 6% (10/16) slept in the farms and 3% (5/162) worked in the rubber plantations. One person spent the whole night in the secondary forest. About 77% (114/148) of the non-rubber workers visited the rubber plantations at least once every month (range in age from one to 96 years) to help with maintenance of the plantation area (cutting undergrowth and clearing fallen trees), to collect fire wood, and to collect food such as mushrooms, insects, and edible plants. More than 90% (148/162) of participants had insecticide-treated bed nets in their houses. Furthermore, a total of 34% (55/162) of respondents used methods to protect themselves against mosquitoes when outdoors, with 60% (33/55) using mosquito coils and 35% (19/55) using the repellent N,N-Diethyl-meta-toluamide (DEET). About 7% (4/55) of participants said they wore long sleeves to protect against mosquito bites and 2% (1/55) mentioned the use of lemongrass.

### Human behavioral typologies

We identified four distinct behavioral typologies: (1) villagers that visit the forest during the day, (2) villagers that work in the rubber plantations, (3) migrant workers that live and work in the rubber plantations, and (4) villagers that stay in the village.

#### Villagers that visit the forest during the day

**Fig 2:**
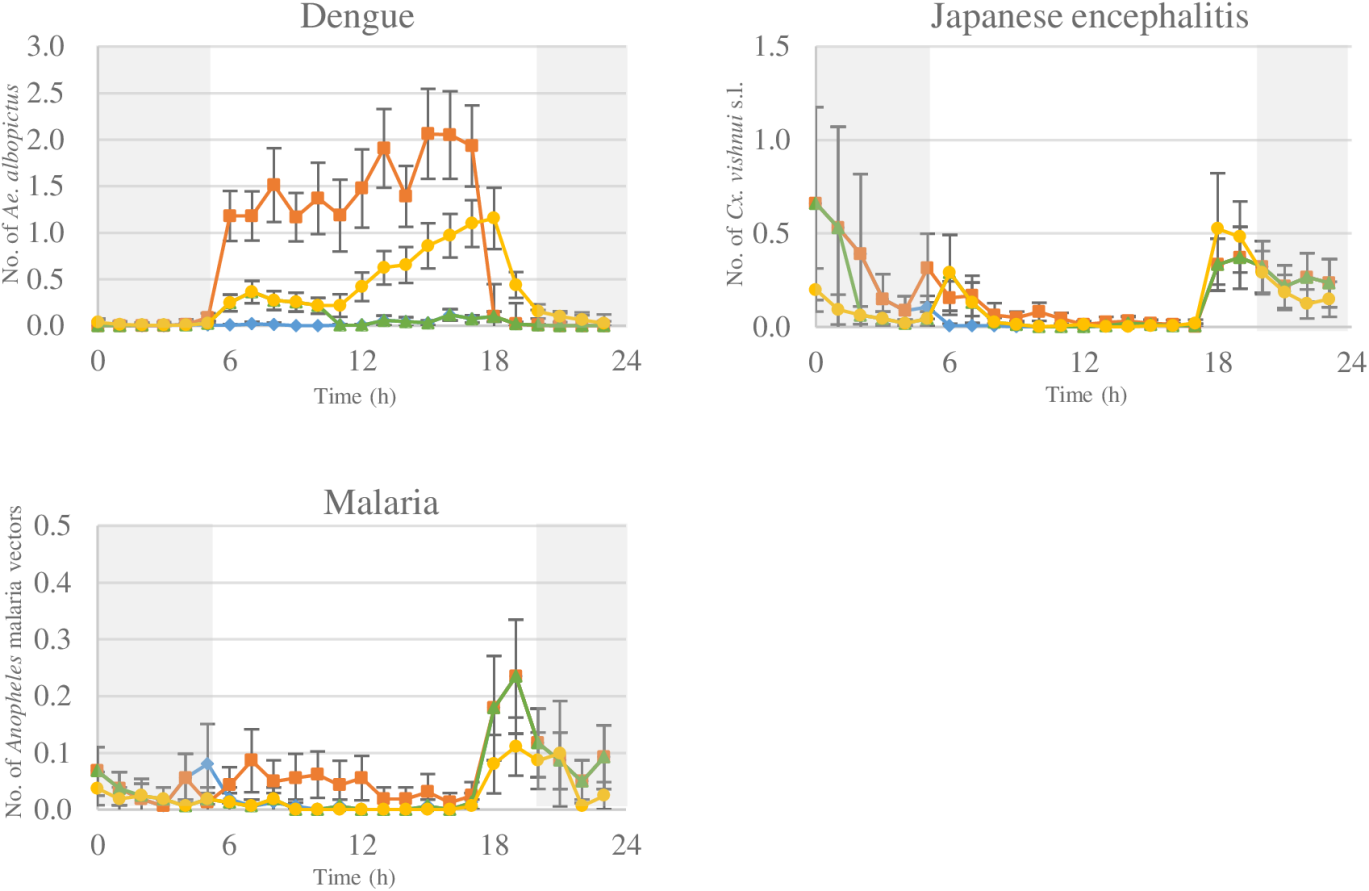
The average hourly exposure to female *Aedes albopictus* (dengue vector), *Culex vishnui* s.l. (Japanese encephalitis vector), and *Anopheles* malaria vectors for the different typologies, (━■━ villagers that visit the forest during the day from 5.00 to 17.00 h, ━▲━ villagers that work in the rubber plantations, ━●━ migrant workers that live and work in the rubber plantations, ━♦━ villager that stays in the village) with the possible use of bed nets indicated from 20.00 h to 5.00 h with █. All including 95% confidence interval.

In this typology, villagers visit the forest during the day from 05.00 h to 17.00 h and sleep in the village at night. Exposure to *Ae*. *albopictus* is highest in the secondary forests during the day from 06.00 h to 17.00 h (Fig 1). Therefore, risk of exposure to *Ae*. *albopictus* is 36 times greater when villagers visit the forest during the day (Fig 2, Table 3). *Culex vishnui* s.l. exposure is also greater when visiting the forest during the day, although highest exposure still occurs in the villages after returning from the forest (Fig 2, Table 3). Exposure to malaria vectors is 1.3 times higher in the forest (Table 3). Risk of exposure to dengue, JE, and malaria vectors is higher for villagers that visited the secondary forests than those that stayed in the village.

**Table 3:** The daily risk of exposure to vectors for people in different human behavior typologies.

	Dengue vector exposure risk			Japanese encephalitis vector exposure risk			Malaria vector exposure risk		
Exposure per 24 hrs	*Ae*. *albopictus* exposure (95% CI)	OR (95% CI)	*P*	*Cx*. *vishnui* s.l. exposure (95% CI)	OR (95% CI)	*P*	Malaria vectors exposure (95% CI)	OR (95% CI)	*P*
**Villagers that visit the forest during the day**	16.8 (14.1–19.4)	36.0 (24.6–52.6)	<0.001*	4.5 (2.9–6.2)	1.4 (1.2–1.7)	<0.001*	1.4 (1.1–1.8)	1.3 (1.2–1.4)	<0.001*
**Villagers that work in the rubber plantations**	1.6 (1.3–2.0)	3.2 (2.3–4.5)	<0.001*	3.6 (2.3–5.0)	1.0 (0.9–1.0)	0.357	1.0 (0.7–1.3)	0.9 (0.8–1.0)	0.062
**Migrant workers that live and work in the rubber plantations**	8.2 (7.0–9.5)	16.2 (11.5–22.9)	<0.001*	2.7 (1.8–3.7)	0.8 (0.6–1.1)	0.195	0.6 (0.4–0.8)	0.6 (0.4–1.0)	0.037*
**Villagers that stay in the village**	0.5 (0.4–0.7)	1		3.7 (2.1–5.4)	1		1.1(0.8–1.4)	1	

Results are shown using generalized estimating equations with odds ratio (OR) and 95% confidence interval (CI).

*significantly different, P<0.05

#### Villagers that work in the rubber plantations

Rubber workers that live in the villages and work in the rubber plantations at night from 02.00 h to 10.00 h are exposed to both village and rubber plantation mosquitoes. Highest *Ae*. *albopictus* exposure occurs when working in the plantation, with peak exposure from 06.00 to 10.00 h (Fig 2). Working in the plantations thus increases dengue vector exposure risk more than three times compared to staying in the village (Table 3). Risk of *Cx*. *vishnui* s.l. exposure is highest when rubber workers are resting in the village with presence in rubber plantations not increasing risk (Fig 2, Table 3). Similarly, malaria vector exposure risk does not increase when rubber plantations are visited at night (Fig 2, Table 3). Compared to remaining in the village, working in the plantations increases the risk of dengue vector exposure, but not for JE or malaria.

#### Migrant workers that live and work in the rubber plantations

Migrant workers that live and work in the rubber plantations are only exposed to mosquitoes present in the mature rubber plantations. Here the risk of dengue vector exposure is 16 fold higher than staying in the village (Fig 2, Table 3). However, rubber workers living in the plantations are exposed to similar number of *Cx*. *vishnui* s.l. mosquitoes and slightly fewer malaria vectors than those remaining in the villages (Fig 2, Table 3). Living and working in the rubber plantations increases risk of dengue vector exposure and decreases risk of malaria vector exposure, compared to villagers staying in the village, while JE vector exposure remains the same.

#### Villagers that stay in village

These individuals are only exposed to mosquitoes present in the village, with peak biting at dusk from 18.00 to 19.00 h (Fig 2). Exposure to mosquitoes is generally low with three *Cx*. *vishnui* s.l., one malaria vector, and 0.5 *Ae*. *albopictus* mosquitoes captured each day (Fig 2, Table 3). When a bed net is used during the night from 20.00 to 5.00 h, exposure to JE vectors can decrease to less than one mosquito exposure every 24 hrs and halve malaria vector exposure (Fig 2). Generally villagers that stay in the village are at low risk of exposure to JE and malaria vectors, with very low risk of exposure to dengue vectors.

### Basic reproduction number for mosquito-borne diseases

#### Mosquito survival

During 42 nights of collection, a total of 1,048 *Ae*. *albopictus* mosquitoes and 82 *Anopheles* malaria vectors (*An*. *aitkenii* group, *An*. *dirus* s.l., *An*. *barbirostris* s.l., *An*. *epiroticus* (Linton & Harbach), *An*. *hodgkini* (Reid), *An*. *maculatus* s.l., *An*. *minimus* s.l., *An*. *tesselatus* (Theobald) and *An*. *umbrosus* s.l.) were dissected. Twenty six *Ae*. *albopictus* and seven *Anopheles* malaria vectors could not be dissected successfully for identification of parity. In general the parity rate was high with long-lived vectors present in all habitats (S3 Table). For *An*. *maculatus* s.l., 34 samples were collected in the four different habitats of which 31 were parous (91.2%). For *An*. *minimus* s.l., 18 samples were collected of which 17 were parous (94.4%) and for *An*. *dirus* s.l., 14 samples were collected, of which eight were parous (57.1%).

#### Basic reproduction number for dengue

The R_0_ for the dengue vector *Ae*. *albopictus* was calculated using the average number of *Ae*. *albopictus* bites per person per day (ma) in the different habitats (S4 Table). The R_0_ was considerably higher than one for all natural and man-made forest habitats during both the rainy season and dry season, and considerably lower than one for the villages (Table 4). The R_0_ was highest in the secondary forests and second highest in the mature rubber plantations. Of the three forest habitats, the R_0_ was lowest in the immature rubber plantations.

**Table 4:** The basic reproductive number (R_0_) for dengue vector *Ae*. *albopictus* in the secondary forest, mature rubber plantation, immature rubber plantation, and village habitats during the rainy season and dry season.

	Secondary forest	Mature rubber plantation	Immature rubber plantation	Village
**Rainy season**	42.0	18.8	9.5	0.06
**Dry season**	10.6	2.8	1.5	0.01

#### Basic reproductive number for malaria

The R_0_ for malaria was calculated using the average number of bites per person per day (ma) for the different malaria vectors in each of the different habitats (S4 Table). Parity data of *An*. *maculatus* s.l., *An*. *minimus* s.l., and *An*. *dirus* s.l. were used separately. All habitats exhibited high malaria R_0_ during both the rainy season and dry season, with similar outcomes for *P*. *falciparum* and *P*. *vivax* (Table 5). Both *An*. *maculatus* s.l. and *An*. *minimus* s.l. are important malaria vectors in the study sites, whilst *An*. *dirus* s.l. is not.

**Table 5:** The basic reproductive number for *P*. *falciparum* and *P*. *vivax* malaria parasites calculated for the different vectors in the different habitats during the rainy season and dry season.

	Malaria parasite	Malaria vector	Secondary forest	Mature rubber plantation	Immature rubber plantation	Village
**Rainy season**	***P*. *falciparum***	***An*. *maculatus* s.l.**	28.6	16.6	64.0	28.6
		***An*. *minimus* s.l.**	8.3	2.8	6.9	42.8
		***An*. *dirus* s.l.**	0.2	0.1	0.5	0
	***P*. *vivax***	***An*. *maculatus* s.l.**	31.2	18.1	69.8	31.2
		***An*. *minimus* s.l.**	8.8	2.9	7.4	45.7
		***A*. *dirus* s.l.**	0.3	0.2	0.7	0
**Dry season**	***P*. *falciparum***	***An*. *maculatus* s.l.**	13.1	22.1	39.2	11.4
		***An*. *minimus* s.l.**	18.1	36.1	41.6	84.9
		***An*. *dirus* s.l.**	0.03	0.2	0.5	0.02
	***P*. *vivax***	***An*. *maculatus* s.l.**	14.9	25.2	44.8	13.1
		***An*. *minimus* s.l.**	19.3	38.5	44.3	90.6
		***A*. *dirus* s.l.**	0.05	0.3	1.0	0.05

## Discussion

We assessed how human behavior changes the risk of exposure to mosquito-borne diseases in rural parts of northern Lao PDR. This study shows that the greatest risk is associated with visiting secondary forest during the day; increasing the risk of dengue, JE, and malaria. Working in the rubber plantations also increases the risk of dengue, which is exacerbated when workers both live and work in the plantations. However, staying in the rubber plantations did not increase risk of exposure to JE vectors and decreased risk of exposure to malaria vectors. Our estimates of R_0_ show that the risk of dengue outbreaks in secondary forests, mature rubber plantations, and immature rubber plantations is extremely high, largely because of the high survival of the vector, *Ae*. *albopictus*. The villages are relative sanctuaries with values of R_0_ considerably less than 1, indicating that the transmission of dengue would not be maintained. The R_0_ estimates also showed that the risk of malaria outbreaks in all investigated habitats is very high, with the most important malaria vector in the rainy season being *An*. *maculatus* s.l. and in the dry season, *An*. *minimus* s.l.

Dengue is a sylvatic disease that has been spread from the forest to rural and urban areas by the highly adaptable vector *Ae*. *albopictus*, that has readily colonized a variety of different rural habitats [58, 59]. In this study, we found a substantial risk of *Ae*. *albopictus* exposure and consequently risk of dengue infection in the natural forests and rubber plantations, compared with the villages. According to the behavioral analysis, both the natural and man-made forests are regularly visited by villagers. It therefore seems likely that the forest and plantation habitats are where dengue transmission occurs. As dengue is endemic in our study area and malaria is not, rubber workers should be encouraged to live in the villages instead of plantations. This is especially important for migrant rubber plantation workers, as presence in the village increases knowledge on diseases and lowers the threshold to get treatment. Worryingly, dengue vector control in the country is presently focused in the villages where the risk of transmission is low. There is therefore a clear need to broaden the control efforts to protect people when entering the surrounding forest and rubber plantations. In future studies, the presence of dengue infections in *Ae*. *albopictus* needs to be molecularly confirmed.

Villagers that visit the secondary forests during the day are exposed to a higher number of JE vectors than when staying in the village. Japanese encephalitis infection risk is dependent on the presence of water birds, the reservoir hosts, and pigs, the amplifying host. Although there are pigs in the forests, there are considerably more water birds and pigs within and close to the villages, increasing the risk of JE infections in the villages. It is therefore important to take the local dynamics of the disease pathogens into account.

Rubber workers that live in the villages are exposed to similar numbers of malaria vectors as the villagers staying at home, with the risk of malaria exposure dropping when workers both live and work in the rubber plantations. This is contrary to earlier suggestions from Thailand that rubber tapping activity increases exposure to malaria vectors [13]. Working in the rubber plantations at night from 02.00 to 10.00 h is not a risky behavior for malaria vector exposure in this study area, due to the early evening host seeking behavior of the malaria vectors *An*. *maculatus* s.l. and *An*. *minimus* s.l. However, the high R_0_ of malaria identified for all habitats does imply that if a malaria-infected person moves into the rubber plantations, the potential for a large number of new infections would arise, transmitted by *An*. *maculatus* s.l. and *An*. *minimus* s.l. We identified two ways in which malaria transmission could occur in the study area. Firstly, we found that local villagers often migrate to find temporary work in other areas of SEA. These migrant workers could be infected by malaria parasites in other regions and carry the parasites back to their own village. Secondly, many of the rubber plantations workers that live in the plantations are migrant workers that only live in the plantations during the rainy season. These migrant workers could introduce malaria parasites from other areas in SEA to the rubber plantation areas. In this study we have shown that the rubber plantations are visited regularly by the local population, indicating that the pathogens established in the rubber plantations could easily spread to the villages. Although malaria is currently not endemic in the study area, if malaria parasites are introduced, all necessary factors are present for an outbreak, and the establishment of malaria. Monitoring the malaria disease presence is thus essential in both the local population and migrant workers. Future entomological studies in the area should focus on the dissection of putative malaria vectors for the identification of sporozoites and oocysts, and focus on the molecular identification of malaria parasites, including the possible presence of *Plasmodium knowlesi*.

Mathematical models simplify the complexity of natural systems. The R_0_ calculations in this paper are no exception. Our models do not consider the dynamics of the larval stages of the mosquitoes, spatial heterogeneity, interrupted feeding of *Ae*. *albopictus*, the vertical and sexual transmission of dengue viruses, nor the immune status of the population. The high basic reproduction numbers found in this study reflects the extraordinarily high mosquito survival rates calculated in this study, often exceeding 90%.

Including human behavioral patterns is important for appropriate recommendations on disease control [60]. There is a lack of suitable methods to measure human behavior, especially on an individual scale, with limits to the predictability of human mobility [61–63]. There are a number of techniques commonly used to capture human movement, such as GPS tracking systems [64, 65], cellular phones [66] and photo voice [67]. In this study we used a combination of PRA’s and surveys to collect human behavior data, which is novel for vector-borne disease studies. The PRA’s and surveys do not result in detailed quantitative information. Both methods are sensitive to memory decay, social desirability, and other biases. Yet the two methods combined allowed us to describe broad patterns of human behavior and relate risk of vector-borne infections to villagers and rubber workers behavior.

Identifying risky behaviors should help explain the heterogeneous pattern of vector-borne diseases, and result in more targeted disease control [61, 68–72]. Currently mosquito control in Lao PDR focusses on the distribution of long-lasting insecticidal nets (LLINs), indoor residual spraying (IRS) and larval source management (LSM) in the villages. The current control strategies are insufficient to control vector-borne diseases, with dengue and malaria outbreaks still occurring regularly. This study has highlighted the importance of secondary forest and rubber plantations in the mosquito-control strategies, specifically for the control of dengue. As in our study area, dengue is an important endemic disease and malaria is not, rubber workers could be encouraged to live in the villages, where dengue vector exposure is lower. Mosquito-control in rubber plantations should focus on the rubber worker houses inside the plantations and on outdoor control. For control in rubber plantation houses, similar methods can be used as in the villages; such as using LLINs, spatial repellents, and screening of houses [12]. For outdoor control, both personal protection and LSM is necessary. Personal protection methods should include motivating rubber workers to wear long-sleeved clothing and closed shoes when in the plantation. Additionally, insecticide-treated clothing, insecticide emanators, and portable insecticide coils could be used for personal protection [12]. However, these personal protection methods need to be further investigated to identify if vector-borne disease cases can be prevented. Rubber plantations provide a plethora of potential breeding sites including latex-collection cups [73–75]. Larval control in rubber plantations can therefore be achieved by draining the latex collection cups by turning them upside down. In forested areas, mosquito control is more challenging than the rubber plantation areas. Particularly larval control is difficult to implement in the natural forests due to the vastness and diversity of breeding sites, and the high biodiversity of other insects present. Emphasis should therefore be on personal protection methods, which are similar to the rubber workers. Additionally, insecticide treated hammocks could be used when staying in the forests overnight [76–78].

## Conclusion

This study demonstrates that entering secondary forest or rubber plantations represents a higher risk of dengue vector exposure than staying in the villages, where current vector control is focused. As rubber workers spend a substantial amount of time in the plantations, this increases their risk of dengue vector exposure compared to villagers who irregularly visit the natural forests or remain in the village. Rubber workers could be encouraged to live in the villages instead of the rubber plantations. Additionally, JE and malaria vector risk increases when visiting the forests during the day, but does not increase when working and living in the rubber plantations. This study highlights the importance of understanding human behavior in order to identify risky behaviors. Specifically, it demonstrates the necessity of broadening current vector control activities to include rubber plantations.

## Supporting information

S1 Table Summary of rapid participatory rural appraisals on hourly variables. Summary of data obtained from the rapid participatory rural appraisals on the daily intensity of mosquito and human activity in different habitats.
(DOCX)

S2 Table Summary of rapid participatory rural appraisals on monthly variables. Summary of data obtained from the rapid participatory rural appraisals on the monthly intensity of environmental variables, mosquito activity, and human activity.
(DOCX)

S3 Table Summary of the parity rate. The parity rate of the dengue vector *Ae*. *albopictus* and of the putative malaria vectors in the secondary forests, mature rubber plantations, immature rubber plantations, and villages.
(DOCX)

S4 Table Mosquito bites per person per day. The average number of mosquito bites per person per day (ma) for the important vector species *Ae*. *albopictus*, *An*. *maculatus* s.l., *An*. *minimus* s.l., and *An*. *dirus* s.l. in the secondary forest, mature rubber plantation, immature rubber plantation and village habitats during the rainy season and dry season.
(DOCX)
