# Supplementary material for: Risk of exposure to potential vector mosquitoes for rural workers in Northern Lao PDR
Source: PLoS Negl Trop Dis. 2017 Jul 25;11(7):e0005802. doi: 10.1371/journal.pntd.0005802 (PMC5544251; doi:10.1371/journal.pntd.0005802)
Supplement: S1 Table — Summary of data obtained from the rapid participatory rural appraisals on the daily intensity of mosquito and human activity in different habitats. (DOCX) [file pntd.0005802.s001.docx]

**S1** **Table summary of rapid participatory rural appraisals on hourly variables**; Summary of data obtained from the rapid participatory rural appraisals on the daily intensity of mosquito and human activity in different habitats

| **Time** | **Periods of mosquito activity** | | | **Periods of human activity** | | | | |
| --- | --- | --- | --- | --- | --- | --- | --- | --- |
|  | **Secondary forests** | **Mature rubber plantations** | **villages** | **Secondary forests** | **Mature rubber plantations** | | **Villages°** | **Rice fields** |
|  |  |  |  |  | **Tapping** | **Latex collection** |  |  |
| **7.00** | **** | *** | * | High activity |  | High activity | Low activity | High activity |
| **8.00** | **** | * | * |  |  |  |  |  |
| **9.00** | **** | * |  |  |  |  |  |  |
| **10.00** | **** | * |  |  |  |  |  |  |
| **11.00** | **** | * |  |  |  | Low activity |  |  |
| **12.00** | **** | * |  |  |  |  |  |  |
| **13.00** | **** | * |  |  |  |  |  |  |
| **14.00** | **** | * |  |  |  |  |  |  |
| **15.00** | **** | * |  |  |  |  |  |  |
| **16.00** | **** | * |  |  |  |  |  |  |
| **17.00** | **** | ** | * |  |  |  |  |  |
| **18.00** | ***** | **** | ***** | Low activity |  |  | High activity |  |
| **19.00** | ***** | *** | ***** |  |  |  |  |  |
| **20.00** | ***** | ** | **** |  |  |  |  |  |
| **21.00** | ** | ** | *** |  |  |  |  |  |
| **22.00** | ** | ** | *** |  |  |  |  |  |
| **23.00** | ** | ** | ** |  |  |  |  |  |
| **24.00** | ** | ** | * |  |  |  |  |  |
| **1.00** | ** | ** | * |  |  |  |  |  |
| **2.00** | ** | ** | * |  | High activity |  | Low activity |  |
| **3.00** | ** | ** | * |  |  |  |  |  |
| **4.00** | ** | ** | * |  |  |  |  |  |
| **5.00** | ** | *** | ** |  |  |  |  |  |
| **6.00** | ***** | **** | **** |  |  |  |  |  |

*No information were collected on mosquito and human activity in immature rubber plantations due to the low and irregular activity of villagers and rubber workers in these habitats* ** Intensity symbol for the different variables according to the experience of the local villagers and rubber workers from none to five °The village behavior is derived from when villagers are not in the secondary forests, rubber plantations and rice fields.*
