# Supplementary material for: Risk of exposure to potential vector mosquitoes for rural workers in Northern Lao PDR
Source: PLoS Negl Trop Dis. 2017 Jul 25;11(7):e0005802. doi: 10.1371/journal.pntd.0005802 (PMC5544251; doi:10.1371/journal.pntd.0005802)
Supplement: S2 Table — Summary of data obtained from the rapid participatory rural appraisals on the monthly intensity of environmental variables, mosquito activity, and human activity. (DOCX) [file pntd.0005802.s002.docx]

**S2 Table summary of rapid participatory rural appraisals on monthly variables**; Summary of data obtained from the rapid participatory rural appraisals on the monthly intensity of environmental variables, mosquito activity, and human activity

|  | **Jan** | **Feb** | **Mar** | **Apr** | **May** | **Jun** | **Jul** | **Aug** | **Sep** | **Oct** | **Nov** | **Dec** |
| --- | --- | --- | --- | --- | --- | --- | --- | --- | --- | --- | --- | --- |
| **Rainfall** |  |  |  |  | ** | *** | ***** | ***** | *** | ** | * |  |
| **Temperature** |  |  | *** | ***** | ***** | **** | *** | *** | *** | * |  |  |
| **Mosquito population** |  | * | * | ** | *** | **** | ***** | ***** | ***** | **** | *** | ** |
| **Villagers feeling unwell** | * |  |  |  |  |  | * | * | * | * | ** | * |
| **Migration** | ** | ** |  |  |  |  |  |  |  |  |  | * |
| **Villagers visit forests** |  |  | *** | *** | *** | **** | ***** | ***** | **** | **** |  |  |
| **Rubber tapping** |  |  |  |  | (End of April - Nov) | | | | | | |  |
| **Rice production** |  |  |  |  | Seedling | | Growing | | | Harvest | |  |

** Intensity symbol for the different variables according to the experience of the local villagers and rubber workers from one to five*
