# Supplementary material for: Risk of exposure to potential vector mosquitoes for rural workers in Northern Lao PDR
Source: PLoS Negl Trop Dis. 2017 Jul 25;11(7):e0005802. doi: 10.1371/journal.pntd.0005802 (PMC5544251; doi:10.1371/journal.pntd.0005802)
Supplement: S3 Table — The parity rate of the dengue vector Ae. albopictus and of the putative malaria vectors in the secondary forests, mature rubber plantations, immature rubber plantations, and villages. (DOCX) [file pntd.0005802.s003.docx]

**S3 Table summary of the parity rate;** The parity rate of the dengue vector *Ae. albopictus* and of the putative malaria vectors in the secondary forests, mature rubber plantations, immature rubber plantations, and villages

| **Habitat** | **Vector species** | **Parity number** | **Parity rate**  **(95 % CI)** |
| --- | --- | --- | --- |
| **Secondary forests** | *Ae. albopictus* | 406/447 | 91 % |
|  | *Anopheles* malaria vectors | 13/14 | 93 % |
| **Mature rubber plantations** | *Ae. albopictus* | 309/327 | 92 % |
|  | *Anopheles* malaria vectors | 5/10 | 50 % |
| **Immature rubber plantations** | *Ae. albopictus* | 234/269 | 87 % |
|  | *Anopheles* malaria vectors | 8/23 | 35 % |
| **Villages** | *Ae. albopictus* | 3/5 | 60 % |
|  | *Anopheles* malaria vectors | 33/35 | 94 % |
| **Total** | *Ae. albopictus* | 953/1048 | 91 % (58 - 100) |
|  | *Anopheles* malaria vectors | 59/82 | 72 % (20 - 100) |

*Results are shown for the parity rate, which is the proportion of parous mosquitoes compared to the total number of mosquitoes successfully dissected. The 95 % confidence interval (CI) is given for the total number of parous mosquitoes.*
