# Supplementary material for: Risk of exposure to potential vector mosquitoes for rural workers in Northern Lao PDR
Source: PLoS Negl Trop Dis. 2017 Jul 25;11(7):e0005802. doi: 10.1371/journal.pntd.0005802 (PMC5544251; doi:10.1371/journal.pntd.0005802)
Supplement: S4 Table — The average number of mosquito bites per person per day (ma) for the important vector species Ae. albopictus, An. maculatus s.l., An. minimus s.l., and An. dirus s.l. in the secondary forest, mature rubber plantation, immature rubber plantation and village habitats during the rainy season and dry season. (DOCX) [file pntd.0005802.s004.docx]

**S4 Table mosquito bites per person per day;** The average number of mosquito bites per person per day (ma) for the important vector species *Ae. albopictus*, *An. maculatus* s.l., *An. minimus* s.l. and *An. dirus* s.l. in the secondary forest, mature rubber plantation, immature rubber plantation and village habitats during the rainy season and dry season

|  | Rainy season | | | | Dry season | | | |
| --- | --- | --- | --- | --- | --- | --- | --- | --- |
| **Vector species** | **Secondary forest** | **Mature rubber plantation** | **Immature rubber plantation** | **Village** | **Secondary forest** | **Mature rubber plantation** | **Immature rubber plantation** | **Village** |
| ***Ae. albopictus*** | 33.3 | 13.1 | 12.2 | 0.8 | 8.9 | 2.1 | 2.1 | 0.2 |
| ***An. maculatus* s.l.** | 0.4 | 0.3 | 0.7 | 0.4 | 0.2 | 0.4 | 0.7 | 0.2 |
| ***An. minimus* s.l.** | 0.07 | 0.02 | 0.06 | 0.3 | 0.1 | 0.3 | 0.3 | 0.7 |
